# Is 3D-printed Titanium cage a reliable option for 3-level anterior cervical discectomy and fusion in treating degenerative cervical spondylosis?

**DOI:** 10.3389/fsurg.2023.1096080

**Published:** 2023-02-17

**Authors:** Shanxi Wang, Xuan Fang, Yunkun Qu, Rui Lu, Xiaojun Yu, Shaoze Jing, Qing Ding, Chaoxu Liu, Hua Wu, Yang Liu

**Affiliations:** ^1^ Department of Orthopedics, Tongji Hospital, Tongji Medical College, Huazhong University of Science and Technology, Wuhan, China; ^2^ Department of Orthopedics, Third Hospital of Shanxi Medical University, Shanxi Bethune Hospital, Shanxi Academy of Medical Sciences, Tongji Shanxi Hospital, Taiyuan, China

**Keywords:** 3D-printed titanium cage, anterior cervical discectomy and fusion, degenerative cervical spondylosis, 3-level ACDF, subsidence

## Abstract

**Background:**

To assess the clinical and radiographical outcomes of 3-level anterior cervical discectomy and fusion (ACDF) with a 3D-printed titanium cage in treating degenerative cervical spondylosis.

**Methods:**

In this study, 25 patients with degenerative cervical spondylosis who underwent 3-level ACDF using a 3D-printed titanium cage from March 2019 to June 2021 were retrospectively enrolled. The patient-reported outcome measures (PROMs) were evaluated by visual analog scale (VAS) for the neck (VAS-neck) and arm pain (VAS-arm), Neck Disability Index (NDI) score, Japanese Orthopedic Association (JOA) score, SF-12 concise health survey, and the Odom criteria. The radiographical parameters, including C2-C7 lordosis, segmental angle, segmental height, and subsidence, were assessed. The mean duration of follow-up was 25.6 months.

**Results:**

Bony fusion was achieved in all patients (100%). In three patients (12%) mild dysphagia was observed during the follow-up. The VAS-neck, VAS-arm, NDI score, JOA score, SF-12 score, C2-C7 lordosis, and segmental angle improved noticeably at the latest follow-up. Based on the Odom criteria, 22 patients (88%) reported satisfactory (excellent or good). The mean loss of C2-C7 lordosis and segmental angle between the immediate postoperative and the latest follow-up values were 1.6° ± 0.5° and 1.1° ± 0.5°, respectively. The mean subsidence was 0.9 ± 0.6 mm.

**Conclusion:**

In patients with multi-level degenerative cervical spondylosis, 3-level ACDF using the 3D-printed titanium cage can effectively relieve the symptoms, stabilize the spine, and restore segmental height and cervical curvature. It is proven to be a reliable option for patients with 3-level degenerative cervical spondylosis. However, a future comparative study involving a larger population and longer follow-up time may be required to further evaluate the safety, efficacy and outcomes of our preliminary results.

## Background

Anterior cervical discectomy and fusion (ACDF) was first mentioned by Smith and Robinson in 1958. It is considered as a safe and effective method to relieve the symptoms of degenerative cervical spondylosis, a common progressive disease among the older population (1–4). With surgical treatment, the compression to nerve root and spinal cord can be relieved immediately, and the patient-reported outcome measures (PROMs) can be improved noticeably (2, 5, 6).

With the development of anesthetic and surgical techniques, there is an increase in the clinical application of ACDF. Furthermore, it has become a mature and prevalent surgical technique in treating degenerative cervical spondylosis. However, determining the type of fusion method that is the best for acquiring bony fusion remains controversial. Additionally, each fusion method has its proponents and inherent drawbacks (2, 7, 8).

In previous studies, a variety of implants were used to promote intervertebral fusion (9–14). Autograft iliac bone, the first implant used for interbody fusion, was replaced gradually due to its bone resorption, graft collapse, and donor-site complications (8, 9, 15). Although the allograft was designed to avoid donor-site complications, its low fusion rate restricted its application (8). Polyetheretherketone (PEEK) cage was the most commonly used biological substitute (15–17). Unfortunately, it may probably lead to a lack of osseointegration, implant subsidence, and even failure of fusion (18). The 3D-printed titanium cage is a new production. It is not only biocompatible but also resistant to corrosion and compression (11). Meanwhile, the porous structure also promotes bony ingrowth, contributing to bone incorporation (16). Several studies have demonstrated that applications of 3D-printed titanium cages in single-level and two-level ACDF can better facilitate interbody fusion and prevent subsidence without increased complications. Yet, based on the authors' knowledge, only a few studies have reported the applications of 3-level ACDF using 3D-printed titanium cages in treating degenerative cervical spondylosis (11, 15, 16, 19). In this study, the clinical and radiological outcomes of patients who underwent 3-level ACDF with a 3D-printed titanium cage were evaluated.

## Patients and methods

### Study design and patients

This study was authorized and approved by the Ethics Committee of our institution. From March 2019 to June 2021, 25 patients who underwent 3-level ACDF with a 3D-printed titanium cage for the treatment of degenerative cervical spondylosis were retrospectively enrolled. In this study, patients aged at least 18 years with symptomatic degenerative cervical spondylosis were included. All the included patients did not respond to conservative treatment before surgeries.

### Participants' baseline data

Of the 25 participants studied, 15 were female patients (60%). The mean age of the participants was 56.8 ± 6.1 years and their mean BMI was 23.2 ± 2.6 kg/m^2^. Four patients (16%) were smokers, and one patient (4%) had diabetes mellitus. Fourteen patients (56%) presented with radiculopathy symptoms, 10 patients (40%) with medullary symptoms, and one patient (4%) with combined symptoms. The most common operative segment was C4-C7 in 14 patients (56%), followed by C3-C6 in 11 patients (44%). Segmental instability was found in 18 patients (72%), and 28 segments (37%) out of 75 segments exist dynamic instability. The preoperative ASA classification was class I in four patients (16%), class II in 18 patients (72%), and class III in three patients (12%). The mean duration of follow-up was 25.6 ± 7.8 months (Table 1).

**Table 1:** Demographic data of patients (*n* = 25).

Variable	Value
Age (years)	56.8 ± 6.1
Gender (female/male), *n* (%)	15 (60%) / 10 (40%)
BMI (kg/m^2^)	23.2 ± 2.6
Smoker, *n* (%)	4 (16%)
Diabetes mellitus, *n* (%)	1 (4%)
Symptoms, *n* (%)	
Radiculopathy	14 (56%)
Myelopathy	10 (40%)
Combined	1 (4%)
Operative segment, *n* (%)	
C3-C6	11 (44%)
C4-C7	14 (56%)
Segmental instability, n (%)	18 (72%)
ASA status (I/II/III), n (%)	4 (16%) / 18 (72%) / 3 (12%)
Follow-up time (months)	25.6 ± 7.8

### Surgical procedure

All the 3-level ACDF surgeries were operated on by the same senior spine surgeons. Following general anesthesia, the patients were given the supine position with their necks properly extended. The C-arm fluoroscope was used to confirm the location of the lesion segment and a standard right-side transverse incision was made. The skin, subcutaneous tissue, and platysma were dissected layer by layer until the front of the cervical vertebral was exposed. Then, the affected intervertebral disc was completely removed with the help of the distractor. After removing the osteophytes and the posterior longitudinal ligament thoroughly, adequate spinal cord and nerve root decompression could be achieved. The cartilaginous endplates were scraped off with a curette. Furthermore, a suitable empty 3D-printed titanium cage was implanted in the intervertebral space and then an anterior cervical plate was fixed. Finally, a drainage tube was retained before closing the incision.

### Postoperative protocol

Following surgery, the symptoms of all patients improved. Postoperative complications, such as dysphagia, hematoma, surgical-site infection, segmental instability, and pseudarthrosis, were recorded. Especially, the dysphagia status was described as none, mild, moderate, and severe (Table 2) (20). All patients were encouraged to wear the cervical collar for eight weeks and take rehabilitation measures early. After that, patients would return to the hospital for clinical and radiological assessments at 1, 2, 3, 6, 12 months, and annually thereafter.

**Table 2:** Bazaz grading system for dysphagia.

Symptom severity	Liquid food	Solid food
None	None	None
Mild	None	Rare
Moderate	None or rare	Occasionally (only with specific food)
Severe	None or rare	Frequent (majority of solids)

### Clinical assessment

All PROMs were recorded preoperatively, postoperatively, and at each follow-up. Some measurement scales in PROMs were used for evaluating the clinical outcomes. The visual analog scale (VAS) was applied to evaluate neck and arm pain levels before and after surgery, including VAS for the neck (VAS-neck) and VAS for the arm (VAS-arm) (21). The Neck Disability Index (NDI) score and Japanese Orthopedic Association (JOA) score were used for assessing the physical and neurological functions preoperatively and postoperatively (22, 23). The SF-12 concise health survey, which selected 12 items from the SF-36 questionnaire, was used to evaluate the general health status and the quality of life comprehensively (24). The minimum clinically important difference (MCID) was considered a threshold for clinical improvement (22). In this study, the MCID values for the above outcome measures were calculated at 2.6 points for VAS-neck pain, 4.1 for VAS-arm pain, 8 for NDI, 2.5 for JOA, 8.5 for SF-12 physical component summary (PCS), and 9.9 for SF-12 mental component summary (MCS) (22, 23, 25, 26). The Odom criteria were proven to be valid and reliable in assessing surgical outcomes and overall patient satisfaction. The following two ratings were used for determining patient satisfaction: satisfactory (excellent and good) or unsatisfactory (fair and poor) (21, 27).

### Radiographic assessment

Radiographs, including the anteroposterior, lateral plain, and flexion-extension radiographs, were collected before surgery, on the first day after surgery, and at each follow-up. The measured parameters included C2-C7 lordosis, segmental angle, and subsidence. The C2-C7 lordosis, also called cervical lordosis, was measured by using the Cobb angle between the lower endplates of C2 and C7. The segmental angle was only limited to fusion levels. Therefore, the measurement approach for this parameter was to use the Cobb angle between the upper endplate of the cephalad and the lower endplate of the caudal vertebrae (2). The subsidence was defined as a change of operative segmental height at the latest follow-up compared with the immediate postoperative height (11). The segmental height was defined as the distance between the midpoint of the superior border of the cephalad-affected vertebral body and the midpoint of the inferior border of the caudal-affected vertebral body. The angle of motion (ROM) ≤4° and translation ≤1.25 mm in the affected levels on flexion-extension images were considered a successful fusion (16).

### Statistical analysis

The SPSS version 20.0 (IBM Corp, USA) was used for statistical analyses. Continuous variables were presented as the mean ± standard deviation (SD) or median (range). Categorical variables were recorded as numbers and percentages. The results of VAS-arm, VAS-neck, NDI, JOA, and SF-12 preoperatively and at the latest follow-up were compared using the Wilcon signed-rank test. The results of C2-C7 lordosis, segmental angle, and segmental height preoperatively and at the latest follow-up were compared using the Paired t-test. A *P*-value of <0.05 was considered to be statistically significant.

## Result

### Operative time, hospital day, and complications

**Figure 1:**
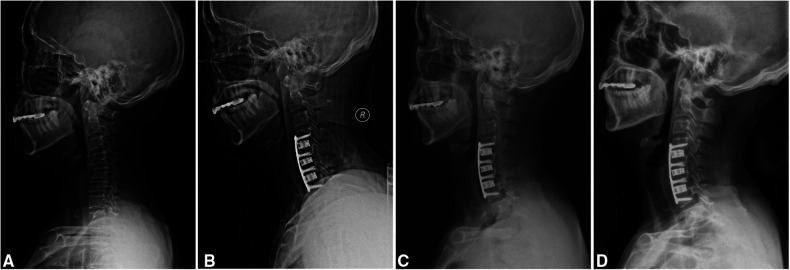
A 60-year-old woman with degenerative cervical spondylosis. (**A**) A preoperatively lateral radiograph in a neutral position. (**B**) This patient was treated with 3-level anterior cervical discectomy and fusion using a 3D-printed titanium cage (C4-7). (**C**) A radiograph at three months post-operatively shows that the implant is in a good position. (**D**) A radiograph at the latest follow-up shows a satisfactory outcome.

The mean operative time of 3-level ACDF was 136.5 ± 7.7 min. Primary healing of incision was achieved in all patients. The median (range) length of hospital stay was 10 days (8–13). The postoperative radiologic data showed that bony fusion was achieved in all patients. Three patients (12%) complained of mild dysphagia during the follow-up, all of which were recovered at latest follow-up. Complications, like hematoma, surgical-site infection, segmental instability, and pseudarthrosis were not noted in any patients after surgery. The typical case is shown in Figure 1.

### Patient-reported outcome measures

**Figure 2:**
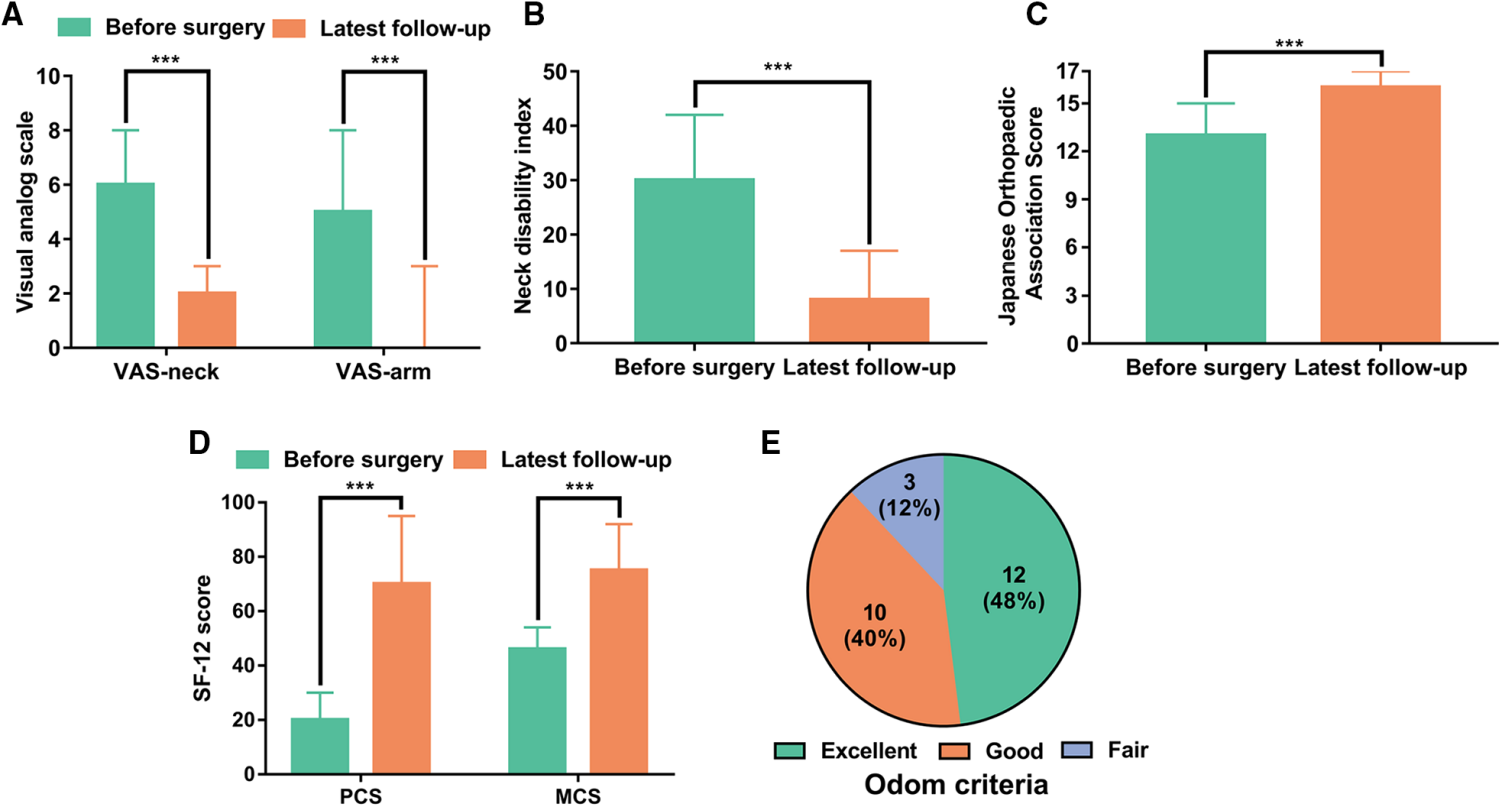
Patient-reported outcome measures before the operation and at the latest follow-up. (**A**) Visual analog scale for neck pain (VAS-neck) and arm pain (VAS-arm). (**B**) Neck Disability Index (NDI) score. (**C**) Japanese Orthopedic Association (JOA) score. (**D**) SF-12 physical component summary (PCS) and SF-12 mental component summary (MCS). (E) Patient satisfaction according to Odom criteria at the latest follow-up. ***P < 0.001.

The median VAS-neck decreased from 6 points (4–8) preoperatively to 2 points (0–3) at the latest follow-up (*P* < 0.001), and the median VAS-arm decreased from 5 points (4–8) preoperatively to 0 point (0–3) at latest follow-up (*P* < 0.001) (Figure 2A). The median NDI decreased from 30 points (20–42) preoperatively to 8 points (3–17) at latest follow-up (*P* < 0.001) (Figure 2B). The median JOA improved from 13 points (7–15) preoperatively to 16 points (14–17) at latest follow-up (*P* < 0.001) (Figure 2C). The median PCS improved from 20 points (0 to 30) preoperatively to 70 points (40 to 95) at latest follow-up (*P* < 0.001), and the median MCS improved from 46 points (17–54) preoperatively to 75 points (42 to 92) at latest follow-up (*P* < 0.001) (Figure 2D). The median differences of VAS arm, VAS neck, NDI, JOA, PCS, and MCS before surgery and at latest follow-up were −4, −5, −22, 3, 50, and 29, respectively, which all reached MCIDs (Table 3). Patient satisfaction was satisfactory (excellent and good) in 22 patients (88%) and unsatisfactory (fair) in three patients (12%) (Figure 2E).

**Table 3:** Comparisons of patient-reported outcome measures between preoperatively and latest follow-up.

Variable	Before surgery (*n* = 25)	Latest follow-up (*n* = 25)	Median difference	*P* value
VAS-neck	6 (4–8)	2 (0–3)	−4	<0.001
VAS-arm	5 (4–8)	0 (0–3)	−5	<0.001
NDI	30 (20–42)	8 (3–17)	−22	<0.001
JOA	13 (7–15)	16 (14–17)	3	<0.001
PCS	20 (0 to 30)	70 (40 to 95)	50	<0.001
MCS	46 (17–54)	75 (42 to 92)	29	<0.001

Data are presented as median (range); VAS, visual analog scale; NDI, neck disability index; JOA, Japanese Orthopaedic Association; PCS, physical component summary; MCS, mental component summary.

### Radiologic assessment outcomes

**Figure 3:**
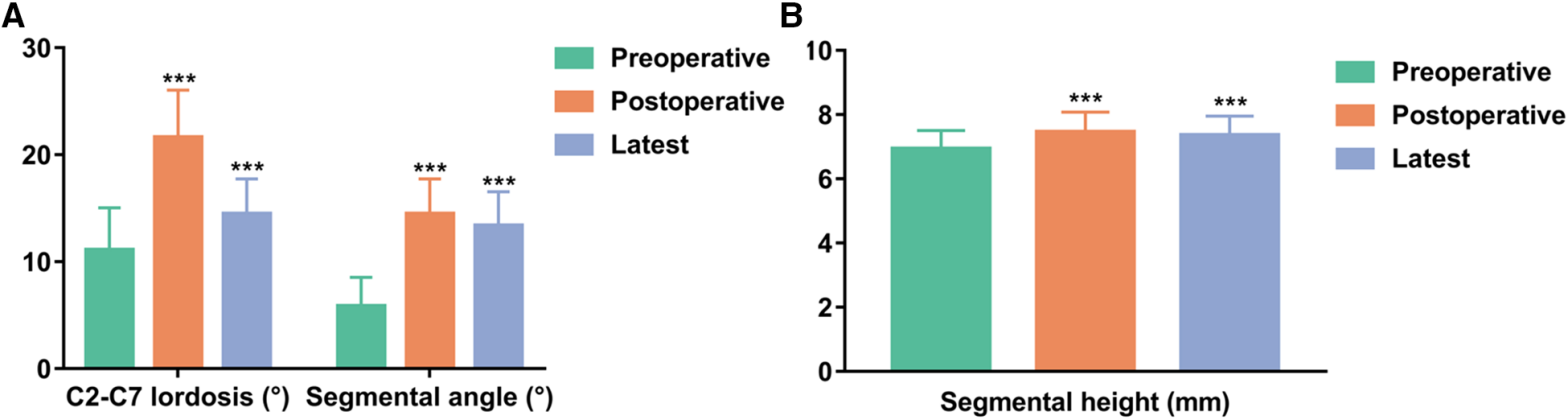
Comparisons of radiologic parameters. (**A**) C2-C7 lordosis and segmental angle. (**B**) Segmental height. ***P < 0.001 vs. preoperative data.

The mean preoperative, postoperative, and latest C2-C7 lordosis were 11.1° ± 4.0°, 21.6° ± 4.4°, and 20.0° ± 4.3°, respectively, and the mean preoperative, postoperative, and latest segmental angles were 5.8° ± 2.7°, 14.5° ± 3.3° and 13.4° ± 3.2°, respectively (Figure 3A). The mean preoperative, postoperative, and latest segmental heights were 69.3 ± 5.7 mm, 74.5 ± 6.2 mm and 73.6 ± 6.0 mm, respectively (Figure 3B). The mean C2-C7 lordosis, segmental angle, and segmental height at latest follow-up were significantly increased compared with preoperative data (*P* < 0.001, *P* < 0.001, *P* < 0.001). The mean loss of C2-C7 lordosis and segmental angle between immediate postoperative and the latest follow-up values were 1.6° ± 0.5° and 1.1° ± 0.5°, respectively. The mean subsidence was 0.9 ± 0.6 mm (Table 4).

**Table 4:** Comparisons of radiologic parameters.

Variable	Radiographic measurements			Comparisons of radiologic parameters	
	Preop.	Postop.	Latest	Loss/Subsidence	Preop. vs. Latest
C2-C7 lordosis (°)	11.1 ± 4.0	21.6 ± 4.4	20.0 ± 4.3	1.6 ± 0.5	*P* < 0.001
Segmental angle (°)	5.8 ± 2.7	14.5 ± 3.3	13.4 ± 3.2	1.1 ± 0.5	*P* < 0.001
Segmental height (mm)	69.3 ± 5.7	74.5 ± 6.2	73.6 ± 6.0	0.9 ± 0.6	*P* < 0.001

Data are presented as mean ± standard deviation.

## Discussion

Degenerative cervical spondylosis has increased in the past decades, exerting a considerable impact on global health (28). Conservative treatments, like oral analgesics, cervical traction, and neck physical therapy can relieve pain and improve neurological function in most patients (4). For patients with surgical indications, ACDF is considered the standard surgery due to its safety and satisfactory clinical results (11). However, as the lesser common procedure, multilevel ACDF is complicated and remains controversial (21). With an increase in the number of fusion segments, the incidence of postoperative complications, like a higher rate of dysphagia, non-union, and subsidence-related complications, are experiencing a rise (5, 10, 29). Wewel et al. found that 3–4 level ACDF could result in pseudarthrosis in nearly half of the patients and had a higher revision rate (10). Another study also reported a higher non-union rate in the 3-level ACDF procedures (30). Hence, an effective fusion technique adopted by surgeons to acquire bony fusion and prevent subsidence-related complications is important.

In the past, autologous iliac bone was regarded as the gold standard for interbody fusion, however, Bolesta et al. reported that the non-union rate was up to 53% in 3-level ACDF using the iliac crest (18, 31). In another related research, the pseudarthrosis rate was 42% in 3-level ACDF using allograft materials (10). At present, the PEEK cage, characterized by cost-efficient and radiolucent, was commonly used for interbody fusion (15, 18). However, its material property was not suitable for bone ingrowth, which was seen as the primary reason for the postoperative non-union (15). A study revealed that the non-union rate in the 3-level ACDF group using PEEK cages for fusion was 14.3%, which was much higher than that of single- and two-level ACDF with PEEK cages (5, 32). The porous structure, promising mechanical properties, and rough surface of the 3D-printed titanium cage could allow bone cell ingrowth, making it easier for interbody fusion and improving the fusion rate (15, 16). In a previous study of 28 patients, the fusion rate in single- or two-level ACDF using a 3D-printed titanium cage was 100% (11). However, scanty information was available in the literature focusing on the fusion rate in 3-level ACDF with a 3D-printed titanium cage. In this study, a 3-level ACDF using a 3D-printed titanium cage in 25 patients with degenerative cervical spondylosis was performed. The mean follow-up time was 25.6 months, and all patients achieved bony fusion. Considering that the fusion rate can be affected by many factors, such as age and smoking, we reviewed the previous literature and found that the patient characteristics included in this study were similar to those in previous studies (5, 10, 33, 34). These data reveal that 3D-printed titanium cage is a feasible choice for interbody fusion.

The fundamental purpose of placing an intervertebral cage after discectomy was to maintain postoperative intervertebral height and cervical lordosis, as well as to prevent the development of subsidence-related complications (8, 35). Fujibayashi et al. presented two types of cage subsidence; transient subsidence, occurring in the early period after surgery, was about 1–3 mm and associated with cage stabilization, while progressive subsidence was associated with non-union (36). Another similar study found that slight subsidence could maintain cervical alignment and lordosis (37). Excessive subsidence could result in segmental kyphosis, adjacent segment degeneration, and failure of fusion (29). One innovative study also revealed that the mild subsidence (1–3 mm) had no effect on clinical outcomes, whereas the severe subsidence (>3 mm) was associated with poor neurological outcomes (38). However, the correlation between cage subsidence and long-term outcomes was still controversial (35, 39). Risk factors leading to cage subsidence were retrospectively studied in the early literature, including increased age, osteopenia, oversized cage, cervical alignment, and use of plate (29). Meanwhile, appropriate cervical curvature was a part of the successful treatment. Chen et al. performed 3-level ACDF using PEEK cage and plate fixation for 26 patients, the loss of cervical lordosis was 2° at 24 months after surgery, and the loss of disc height was about 1.4 mm (5). Louie et al. reported that for a 3-level ACDF using a PEEK cage, the mean subsidence was 1.7 mm after a mean 24.3-month follow-up (40). Achieving interbody fusion in a 3D-printed titanium cage is faster than in a PEEK cage, which can effectively prevent subsidence and loss of cervical lordosis (16). In our study, the loss of cervical lordosis at the latest follow-up was 1.6°, and the mean subsidence was 0.9 mm. Our study indicated that a 3D-printed titanium cage is an effective option for maintaining postoperative intervertebral height and cervical lordosis.

A large number of studies have shown that ACDF can significantly improve the PROMs of patients after surgery (9, 16, 21, 33). Lambrechts et al. reported 1024 patients who underwent ACDF, and all PROMs, including VAS neck and arm pain, NDI, JOA, and SF-12 scores, improved noticeably (41). Arts et al*.* retrospectively reviewed 49 patients who underwent single-level ACDF surgeries. The mean VAS arm and neck pain scores decreased from 56.1 points and 53.2 points preoperatively to 22.2 points and 23.8 points postoperatively at 12 months, respectively. The mean NDI decreased from 41.2 points preoperatively to 19.4 points postoperatively at 12 months (16). In this study, a significant reduction in neck and arm pain was observed. The disability, physical, and neurological functions of the patients showed a noticeable improvement at the latest follow-up compared with the preoperative data, as illustrated by the improvement in the SF-12, NDI, and JOA scoring systems. Moreover, the MCID was used to evaluate the improvement of clinical outcomes, and all PROMs achieved MCID. At the latest follow-up, 88% of patients responded with satisfactory outcomes (excellent or good) based on the Odom criteria.

The most common complication after ACDF is dysphagia (42, 43). Nanda et al*.* performed 3-level ACDF for 25 patients, four of whom had dysphagia in the postoperative period (44). Sun et al*.* compared the clinical outcomes of zero-profile spacer (ZP Group) and plate-cage (PC Group) for 3-level ACDF, 40.7% of patients in ZP Group and 47.1% of patients in PC Group experienced dysphagia at 48 h postoperatively, and 3.7% of patients in ZP Group and 23.5% of patients in PC Group still had dysphagia at 6 months after surgery (45). In another study about 3- or 4-level ACDF using allograft materials, 11% of patients had clinically significant dysphagia at discharge (10). In the present study, three patients (12%) complained of mild dysphagia during the follow-up, all of which were recovered at latest follow-up. Our data preliminarily support the 3D-printed titanium cage as an option for 3-level ACDF in treating degenerative cervical spondylosis with comparable incidence of complications to traditional approaches.

There are also some drawbacks in 3D-printed titanium cage utilization, the major concerns are the fatigue performance and mechanical strength. Although the design of porous structure can promote bone ingrowth and reduce elastic modulus, it may impair the fatigue performance and mechanical strength of implants(11). However, biomechanical assessment revealed better mechanical properties of 3D-printed titanium cage than those of conventional implants, which supports that 3D-printed titanium is a feasible implant for 3-level ACDF (46).

A few limitations were observed in the present study. First, a control group was lacking in this study. Future research should compare the outcomes of the 3D-printed titanium cage with other cages in 3-level ACDF. Second, the number of patients in this study is too small to perform an effective subgroup analysis including more relevant factors, such as osteoporosis and gender. Cervical spondylosis is more common in the elderly, often accompanied by osteoporosis and other diseases. Previous studies have shown that patients with osteoporosis have a lower fusion rate after ACDF (47, 48). Compared with PEEK cage, 3D-printed titanium cage can accelerate the achievement of interbody fusion (16). This may be a feasible option for people with osteoporosis. However, because of the few clinical application of 3D-printed titanium cage, there are no related studies about the application of ACDF using 3D-printed titanium cage in patients with osteoporosis have been reported, and further studies are needed to verify this in the future. At last, a longer duration of follow-up is needed to investigate long-term complications and cervical stabilization. Despite these limitations, the present study remains the first retrospective study evaluating on the efficacy of 3-level ACDF using the 3D-printed cage in treating cervical spondylosis.

## Conclusion

In patients with multi-level degenerative cervical spondylosis, 3-level ACDF using the 3D-printed titanium cage can effectively relieve the symptoms, stabilize the spine, and restore segmental height and cervical curvature. The fusion rate of 100% can be reached. It is proven to be a reliable option for patients with multi-level degenerative cervical spondylosis. However, due to the lack of a corresponding control group and limited sample size, the safety, efficacy and outcomes of our preliminary results have not been fully confirmed. A future comparative study involving a larger population and longer follow-up time may be required to further evaluate the clinical outcomes between the 3D-printed titanium cage and traditional implants in 3-level ACDF. This will further help in supporting the findings of our study.

## Data Availability

The raw data supporting the conclusions of this article will be made available by the authors, without undue reservation.
